# The Use of Telemedicine in the Preoperative Care of Pheochromocytoma: A Systematic Review

**DOI:** 10.7759/cureus.38290

**Published:** 2023-04-29

**Authors:** Abdulhameed Alhazmi, Moeber M Mahzari, Sameerah Alshehri, Abdulaziz Alhazmi

**Affiliations:** 1 Department of Internal Medicine, Jazan University, Jazan, SAU; 2 Department of Medicine, Ministry of the National Guard Health Affairs, Riyadh, SAU; 3 Department of Medicine, College of Medicine, King Saud Bin Abdulaziz University for Health Sciences, Riyadh, SAU; 4 Department of Medicine, Ministry of National Guard Health Affairs, Riyadh, SAU; 5 Department of Population Health, King Abdullah International Medical Research Center, Riyadh, SAU; 6 Department of Pathology, Jazan University, Jazan, SAU

**Keywords:** neuroendocrine, telehealth, telemedicine, covid-19, preoperative, pheochromocytoma

## Abstract

Pheochromocytoma (PCC) is a neuroendocrine tumor that may present with headaches, palpitations, and hypertension, and if left unresected, it can lead to serious complications and fatal cardiac mortality. Adequate preoperative management can decrease the risk of intraoperative complications. In this systematic review, we address and discuss what has been published in the literature about the optimization of pheochromocytoma preoperative care via various types of telemedicine (TM). We searched health research databases PubMed, Medical Literature Analysis and Retrieval System Online (MEDLINE), the Cochrane Library, and Google Scholar for literature on various types of TM employed for PCC preoperative management. We searched peer-reviewed literature in the English language published in the literature until November 5, 2022, using medical subject heading (MeSh) terms in PubMed like "telemedicine" and "pheochromocytoma." We used "telemedicine" or "telehealth" and "pheochromocytoma" in other databases. We considered all types of TM, including synchronous, asynchronous, and remote patient monitoring. Our search yielded five publications in PubMed, 59 results in Google Scholar, and none in the Cochrane Library. After excluding duplicates and evaluating the articles for relevance, five papers were selected for this review. Studies came from the United States and Italy. Findings from these studies suggested safe outcomes and reduced costs compared to what is traditionally followed in physical settings. Overall, this systematic review shows the convenience and safety of TM use for a broad spectrum of patients. Further studies are needed to consolidate these findings. Moreover, guidelines on patients’ selection and procedures for safe and effective TM care for patients with PCC are required.

## Introduction and background

Pheochromocytoma (PCC) is an adrenal medullary tumor that arises from the chromaffin cells, resulting in a high level of catecholamines. Due to the adrenergic effects of catecholamines, PCC manifests clinically with headaches, palpitations, and hypertension (HTN) [1]. PCC, if left untreated, is associated with significant cardiovascular morbidity and end-organ damage, such as cardiac arrhythmias, myocardial infarction, and cerebrovascular accidents [2-3]. The mainstay of PCC treatment is open or laparoscopic surgical resection of the tumor. As surgical resection is a high-risk procedure, it requires appropriate preoperative preparation to minimize excessive catecholamines, adrenergic stimulation, and intraoperative hypertensive crises [2].

Proper patient preparation before the PCC surgery could lower the mortality rate to less than 1% [4]. The cornerstone of preoperative management in such patients is to control blood pressure (BP) and heart rate (HR) and restore intravascular volume. The Endocrine Society guidelines recommend that patients with PCC receive adequate alpha blockade for seven to 14 days before surgery to achieve optimal BP and HR. The suggested target for BP is less than 130/80 mmHg while seated and a systolic BP greater than 90 mmHg while standing, with an HR target of 60-70 beats per minute (BPM) seated and 70-80 BPM standing [2]. Alpha-blockers, such as phenoxybenzamine, are the first-line treatment in these patients; however, calcium channel blockers are an acceptable alternative in cases of mild hypertension [4-5]. Frequent clinical assessment is indicated to ensure proper preoperative preparation and achieve BP and HR targets. This frequent clinical assessment could be a burden on patients and the health care system in areas with limited resources. Therefore, the care of PCC patients via telemedicine (TM) has been used and could be of value to such patients.

TM can be described as the use of medical information exchanged from one site to another via electronic communications to improve a patient’s clinical health status during a remote clinical service [6]. TM's utilization for preoperative management has been conducted for various presurgical procedures such as maxillofacial, trauma, and pediatric surgery [7-9]. A few studies reported the use of TM in preoperative care for PCC. Preoperative preparation and titration of antihypertensive medications through TM have demonstrated less need for preoperative visits [10], a decreased risk of infection [5,11,12], and high safety in the management of complex cases [11,13].

Many studies originated to evaluate the advantages of using TM in preoperative care for PCC. This systematic review aimed to discuss and summarize the available data on the effectiveness and safety profiles of different TM practices in the preoperative management of PCC.

## Review

Methodology

Search Strategy

For this review, two reviewers (A.A. and M.M.) independently searched for relevant articles using health research databases PubMed, Medical Literature Analysis and Retrieval System Online (MEDLINE), the Cochrane Library, and Google Scholar for articles on various types of TM employed for PCC preoperative management. We searched peer-reviewed literature in the English language published until November 5, 2022. We used the following MeSh terms in PubMed: "telemedicine" and "pheochromocytoma." We used the following words in the other databases: "telemedicine" or "telehealth" and "pheochromocytoma.". We considered all types of TM for this systematic review, including synchronous, asynchronous, and remote patient monitoring.

Inclusion and Exclusion Criteria and Outcome Measures

**Figure 1:**
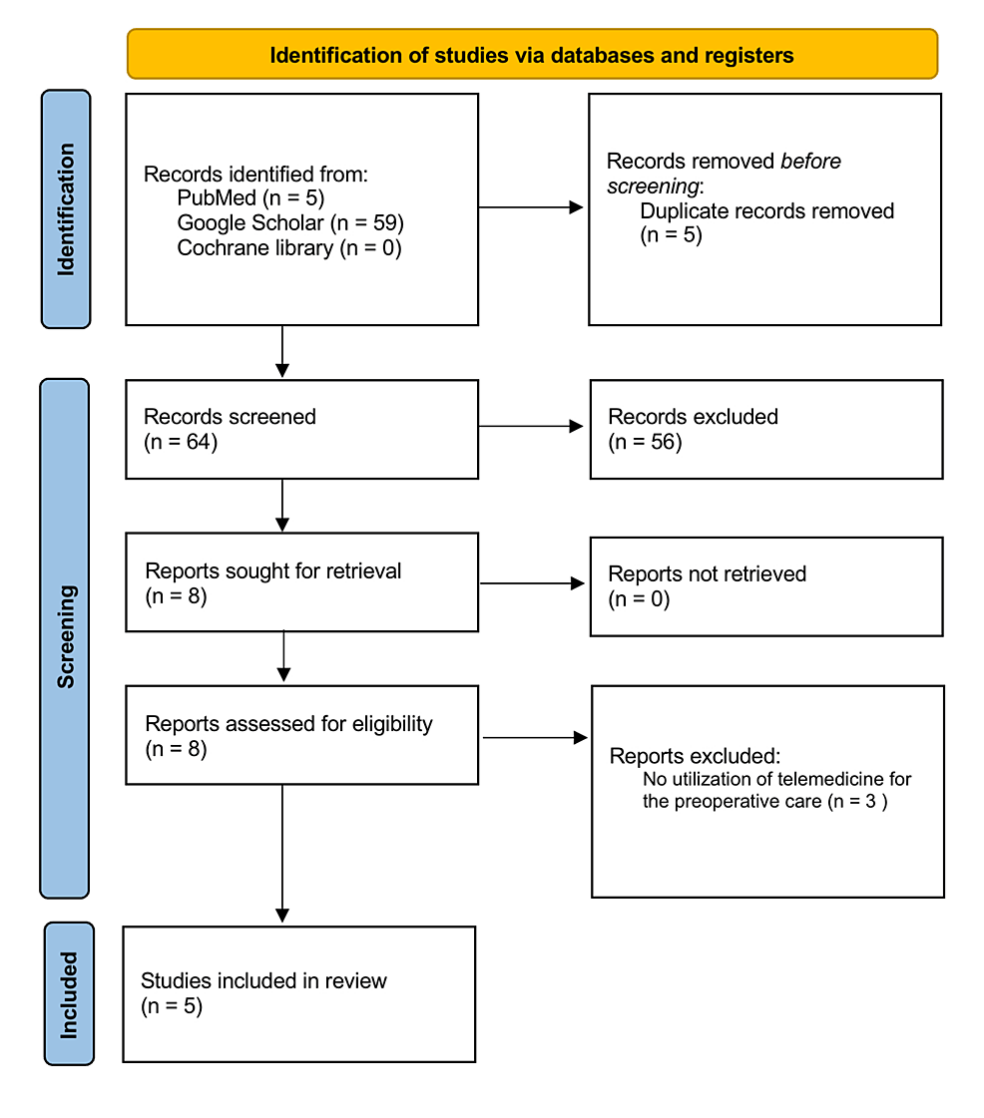
PRISMA flowchart of selection of studies PRISMA: Preferred Reporting Items for Systematic Review and Meta-Analysis

We selected articles that discussed the preoperative management of PCC using telemedicine that have been published in English and provided: (1) a clear diagnosis of PCC; (2) the utilization of TM for preoperative care; and (3) the outcome of the surgery. We excluded articles that were exclusive to consultations or did not contain clinical content. Figure 1 illustrates the Preferred Reporting Items for Systematic Reviews (PRISMA) flow chart of the study selection process. The primary outcome assessed in the systematic review was the success of the surgery, whether it was eventful or uneventful.

Search

Our search yielded five publications in PubMed, 59 results in Google Scholar, and none in the Cochrane Library. After excluding duplicates and evaluating the articles for relevance, five papers were ultimately selected for this review (Table 1). These were: a case-control, a case series, two case reports, and one letter to the editor. Studies came from the United States and Italy.

Data Extraction

We cited the selected articles' details in an Excel spreadsheet to evaluate the methods of each study, modalities utilized, variants reported, results, and limitations of individual studies.

Results

Table 1 summarizes all the included studies. Two of these studies were case reports; one was a case series; one was an original article; and one was a letter to the editor. Most of these reports were from the United States of America and were published in 2020. The majority of the included participants were females aged 17 to 86 years. The comorbid condition with HTN was dominance. Two cases with complex medical histories and pregnancies benefited from special consideration [11,13]. All authors used different approaches to utilizing TM for the initial encounter, where most cases were assessed physically and others chose to use video teleconference TM. Physicians followed their patients via all methods, i.e., synchronously through the phone, asynchronously via email, and with a remote patient, using a mobile phone application. Follow-ups for most patients were confined to twice weekly, excluding the pregnant patient [13], which was daily. The preoperative duration ranges from three weeks to ten months. All articles reported successful, uneventful surgical procedures.

**Table 1:** Characteristics of the included studies * The authors report a successful follow-up with their patient using telemedicine. However, one notable limitation is the lack of specific details about telemedicine encounters. USA: United States of America; F: female; M: male; HTN: hypertension; F/U: follow-up; DM: diabetes mellitus

References	Article type	Country	Date	Gender	Age, in years	Comorbidities	Type of anti-HTN medications	Type of the initial visit	Type of telemedicine follow up	Specific types	Frequency of the follow-up	Duration of f/u before surgery	Postoperative events
Japp EA et al. [5]	Case series	USA	2020	F	60	HTN and prediabetes	Calcium channel-blocker: amlodipine, 7.5 mg daily	Telemedicine/video	Synchronous	Phone	Twice weekly	Seven weeks	Uneventful
				F	67	HTN, type 2 DM, and primary hyperparathyroidism.	Alpha-blocker: terazosin, 1 mg daily; beta-blocker: metoprolol tartrate, 12.5 mg twice daily	Physical	Synchronous	Phone	Twice weekly	12 weeks	Uneventful
				F	84	HTN, dyslipidemia, hypothyroidism, and anxiety	Calcium channel blocker: amlodipine, 2.5 mg daily, and angiotensin-converting-enzyme inhibitor: ramipril, 2.5 mg	Physical	Synchronous	Phone	Twice weekly	18 weeks	Uneventful
Heslin MJ et al. [10]	Case-control	USA	2019	F: 9 M:5 Total of 14 patients	≤55 :8 / >55 : 6	Unavailable	Alpha-blocker (not mentioned)	Physical	Asynchronous	Email	Twice weekly	4.7 weeks (average)	Uneventful
Zampetti B et al. [11]*	Case report	Italy	2021	F	83	History of HTN, dyslipidemia, heart failure, reverse Takotsubo, cardiogenic shock, acute kidney injury, gastroesophageal reflux, autonomous thyroid adenoma, cholecystectomy, and history of breast cancer	Alpha-blocker: doxazosin, 8 mg twice daily, in addition to angiotensin-converting enzyme inhibitor: ramipril and calcium channel-blocker: lercanidipine, beta-blocker: atenolol, and diuretic: furosemide were prescribed	Unavailable	Unavailable	Unavailable	Unavailable	10 months	Uneventful
Yu R [12]	Letter to Editor	USA	2020	M	17	HTN	Alpha-blocker: phenoxybenzamine, 30 mg twice daily; and beta-blocker: metoprolol, 100 mg twice daily.	Telemedicine/Video	Asynchronous	Email	Twice weekly	3 weeks	Uneventful
Saksa D et al. [13]	Case report	USA	2020	F	26	Von Hippel Lindau syndrome and pregnant	Alpha-blocker: terazosin 1-3 mg	Telemedicine/Video	Remote patient monitoring / asynchronous	Mobile phone application	Daily	Unavailable	Uneventful

Discussion

Due to accumulated evidence, this systemic review was conducted to fill the knowledge gap on the usefulness of TM in the preoperative care of PCC. In this systematic review, we used PubMed, MEDLINE, Google Scholar, and the Cochrane Library to maximize the included research that discusses PCC preoperative care using TM. It’s noteworthy that most of the included studies were conducted during the COVID-19 pandemic.

Telemedicine has three major types: synchronous, asynchronous, and remote patient monitoring [14], and these different types are classified according to the timing of information transference. Synchronous TM is called real-time interaction, such as video calls or telephone calls. Asynchronous TM, also known as store-and-forward TM, is about acquiring the data from a sender and then relaying it to a recipient (for example, emails). Remote patient monitoring, as the name implies, uses technological devices to monitor and follow up on the health and clinical signs of patients [14].

The benefits of TM in PCC care have been demonstrated. For example, it is known that alpha-blockers are the primary pharmacological agents to control BP before PCC surgery [2]. However, to reach the targeted BP, the patient usually requires multiple clinic visits for dose titrations [10]. Heslin et al. conducted a case-control study to assess the safety and efficacy of preoperative TM titration of alpha-blockers for 14 patients, compared to a control group of 14 patients who were followed up physically [10]. The authors reported that the TM arm had fewer preoperative appointments and a shorter time to stop the alpha-blockers from initiation of the surgery compared to the control group. Likewise, it was concluded by Saksa et al. [13] and others [10] that TM can avoid the disadvantage of long-distance travel or long driving for physical visits, and the patient in Saksa et al.'s case expressed this fact as TM care saved her two hours’ drive.

On the other hand, TM can decrease the risk of infection, as was observed during the COVID-19 pandemic [5,11,13]. After COVID-19 hit the world, it was necessary to substitute the physical model of care with TM to minimize COVID-19 transmission to the community and healthcare workers. For instance, Japp et al. followed a COVID-positive patient till she got cleared [5].

One question that represents a challenge for physicians is which patient is suitable for TM care. In general, three major factors are important to consider. The first factor is the regulations and health system settings in terms of TM use in health care. The second factor is the availability of adequate infrastructure for proper TM use, such as the availability of required devices and platforms. The third factor is the patient characteristics and eligibility for TM care, which include patient medical history, clinical stability, literacy, and the ability to use TM platforms effectively [14].

The majority of studies did not report legal or technical obstacles in their experiences with patients who accepted TM visits [5,10,11,13]. However, when managing younger patients, a legal guardian's approval will be required, as reported in one article where the author acknowledged that they had to discuss the follow-up with the patient’s mother [12].

Patient characteristics are major determinants where the differences in physicians' perspectives on using TM become evident. TM in preoperative care for PCC is emerging, and consequently, there are no current clinical trials or guidelines that advise whom to consider for a TM visit. Nonetheless, even patients with comorbidities could benefit from TM care. For example, Zampetti et al. reported a patient with PCC and a complicated medical history, including heart failure, cardiogenic shock, and a severe hypertensive crisis. Despite this, the patient was managed successfully via TM with a 10-month follow-up [11]. Another example is a PCC in pregnancy, which represents a high-risk condition requiring intervention before the 24th week to avoid the expected risks to the mother and fetus [15]. With almost entirely TM care, Saksa et al. successfully managed a pregnant lady with PCC, from an initial TM video visit to subsequent remote patient monitoring visits ending with uneventful surgery [13].

Moreover, TM adds flexibility to preoperative follow-ups, which vary from three weeks to 40 weeks [5,10,11,12]. The variation in the time of the preoperative follow-up was reported to be due to the COVID-19 infection in Japp et al.'s second case [5] or waiting for the adrenal hematoma to resolve in Zampetti et al.'s case [11].

The three studies, i.e., Japp et al., Run, and Saksa et al., all used video-synchronous TM for their initial evaluation [5,12,13]. The others used the regular physical encounter for the initial assessment of a new patient. During follow-up, all authors reported that the medication adjustments were based on the reported BP and HR by the patients. Japp et al. used synchronous TM and contacted patients by telephone [5], while Heslin et al. [10] and Run [12] selected asynchronous TM and exchanged data with patients using emails. Regardless of TM type, the authors succeeded in keeping track of their patients twice weekly. Saksa et al. utilized the remote patient monitoring type of TM care [13], where the patient entered BP and HR values via a mobile phone application, and the physician responded accordingly with a management plan in terms of medication titration. This mobile application allowed both parties to exchange secure messages, with follow-up on a daily basis. Given the scarcity of data and the lack of satisfaction studies, it is difficult to identify a favorable TM approach.

However, it is essential to educate the patient about life-threatening complications such as hypertension urgency or emergency, the medications' side effects [10], how to use medical devices such as BP measuring machines [10,12,13], and provide easy access to the appropriate medical care when needed [10,12].

The current systematic review has some limitations. Due to the rarity of PCC, the majority of the included studies are case reports, minimizing the possibility of generalizing the results to a larger population or other nations. Further, there are no cohort studies or clinical trials that can be used to assess the quality and reach a better outcome. Most articles did not report important clinical aspects, such as BP or HR. However, we believe that the benefits of using TM in PCC preoperative care are non-negligible and deserve more evaluation.

## Conclusions

Overall, this systematic review examined the available literature on TM use in PCC care. It shows the convenience and safety of TM use in that all the patients had a successful, uneventful adrenalectomy without reported complications. Further studies are needed to consolidate these findings. Moreover, guidelines on patients’ selection and procedures for safe and effective TM care for patients with PCC are required.
